# Cost‐Effectiveness of a Culturally Adapted Diabetes Self‐Management Education Program for Marshallese Adults With Type 2 Diabetes‐a State‐Transition Markov Analysis

**DOI:** 10.1111/dom.71351

**Published:** 2026-09-17

**Authors:** Daniel Lamprecht‐Carson, Hongmei Wang, Su‐Hsin Chang, Pearl A. McElfish, Jennifer A. Andersen, Tzeyu L. Michaud

**Affiliations:** ^1^ Department of Health Services Research & Administration, College of Public Health University of Nebraska Medical Center Omaha Nebraska USA; ^2^ Division of Public Health Sciences, Department of Surgery Washington University School of Medicine St Louis Missouri USA; ^3^ College of Medicine University of Arkansas for Medical Sciences Northwest Springdale Arkansas USA; ^4^ Institute for Community Health Innovation, College of Medicine, University of Arkansas for Medical Sciences Springdale Arkansas USA; ^5^ Department of Health Promotion, College of Public Health University of Nebraska Medical Center Omaha Nebraska USA; ^6^ Center for Reducing Health Disparities, College of Public Health University of Nebraska Medical Center Omaha Nebraska USA

**Keywords:** cost‐effectiveness, diabetes complications, health economics, type 2 diabetes

## Abstract

**Aim:**

To assess the health and economic consequences of a culturally adapted family‐model diabetes self‐management education (adapted DSME) program for Marshallese adults with type 2 diabetes (T2D) compared with standard DSME and usual care.

**Methods:**

A state‐transition Markov model was constructed to predict costs and quality‐adjusted life years (QALYs) over a 20‐year time horizon from a healthcare sector perspective. Health states reflected T2D‐related complications. Model inputs included intervention costs, complication‐related costs, health state utilities, and transition probabilities. Effectiveness of the adapted and standard DSME programs was defined as the reduction in haemoglobin A1c observed in a 12‐month randomized controlled trial. Cost and health utility parameters were obtained from published literature. Cost‐effectiveness was evaluated using an incremental cost‐effectiveness ratio (ICER) metric. Deterministic and probabilistic sensitivity analyses (PSA) were conducted in which input parameters and willingness‐to‐pay (WTP) thresholds were varied to reflect model uncertainty.

**Results:**

The adapted DSME program was cost‐effective, with an ICER of $3083 per QALY gained, even at the conservative willingness‐to‐pay threshold of $50 000 per QALY. The results were most sensitive to variations in the costs of the adapted DSME program and ongoing dialysis. PSA comparing all three strategies showed the adapted DSME program was the preferred strategy in 87.1% and 93.7% of simulations at WTP thresholds of $50 000 and $100 000 per QALY, respectively.

**Conclusions:**

The culturally adapted DSME program for Marshallese adults with T2D is a cost‐effective intervention over a 20‐year time horizon, although the results should be interpreted with caution because the differences in costs and health outcomes between the intervention strategies were modest. Nevertheless, these findings support the potential value of culturally tailored diabetes education programs and provide evidence to inform reimbursement decisions by public and private health insurers.

## 1 Introduction

The prevalence of type 2 diabetes (T2D) in the United States (U.S.) increased by 2.3% from 2001 to 2023 and affects approximately 38 million people [1]. T2D imposes a substantial financial burden on both patients and the healthcare system. Individuals with T2D are at increased risk of both microvascular and macrovascular complications [2], and diabetes accounts for nearly one‐quarter of total healthcare expenditures in the U.S. [3] Direct medical costs attributable to diabetes exceed $300 billion annually, with average annual medical expenditures of approximately $20000 per individual with T2D [3].

Significant racial and ethnic disparities persist in diabetes prevalence [1]. One population with particularly high rates of T2D is Marshallese Pacific Islanders residing in the U.S. Studies have indicated the prevalence of T2D among Marshallese adults is as high as 38.4%–48.2% [4, 5], substantially higher than the 11.7% diagnosed prevalence among Native Hawaiians or Pacific Islanders and the overall U.S. prevalence of 13.5% [1]. Prior studies have also showed suboptimal diabetes self‐management among Marshallese adults with T2D: only 46.0% reported daily blood glucose monitoring [6], compared with a national estimate of 63.4% [7], and only 47.8% reported receiving an annual foot exam [6], compared with 71.6% nationally [8].

One approach to improve diabetes management is through diabetes self‐management education (DSME) programs. DSME aims to provide individuals with diabetes with the knowledge, skills, and confidence needed to effectively manage their condition. Participation in DSMEs has been associated with several benefits, including reduction in haemoglobin A1c (HbA1c) levels [9, 10], lower rates of diabetes‐related complications [11], decreased all‐cause mortality [12], and improved quality of life [13]. Despite these findings, standard DSME may be relatively ineffective among ethnic minority groups, and DSME programs that are culturally adapted to the specific needs and values of participants may produce better health outcomes [14]. For example, a randomized controlled trial (RCT) conducted in Arkansas among Marshallese adults with T2D showed a 0.9% reduction in HbA1c after 12 months in participants receiving a culturally adapted DSME program, compared to just a 0.1% reduction in those receiving a standard DSME program [15].

Although culturally adapted DSMEs have demonstrated efficacy, the literature evaluating their cost‐effectiveness remains limited [16, 17], especially among minority populations. Examples of such research include a within‐trial cost‐effectiveness analysis (CEA) of a culturally adapted dietary program for Latinas with T2D, where the authors reported a cost of $7723 per unit reduction in HbA1c and body mass index (BMI) over a 6‐month period compared to usual care [16]. In addition, a simulation study with a 20‐year time horizon by Prezio et al. found that a culturally tailored diabetes education and management program, when added to usual care for uninsured Mexican Americans, yielded an additional $355 per quality‐adjusted life year (QALY) gained [17]. Accordingly, the objective of this study was to assess the health and economic consequences of a culturally adapted, family DSME program for Marshallese adults with T2D to inform stakeholders considering the adoption of similar programs in their communities.

## 2 Materials and Methods

### 2.1 Study Design

We applied a state‐transition Markov cohort model with annual cycles to estimate 20‐year health outcomes and costs of Marshallese adults with T2D as a result of the adapted DSME, compared with the standard DSME and usual care, from a healthcare sector perspective. Costs and utilities were discounted annually at a 3% rate [18]. Results were reported following the Consolidated Health Economic Evaluation Reporting Standards guidelines (Table S1) [19].

### 2.2 Study Context

The interventions evaluated in this analysis were drawn from a RCT conducted among Marshallese adults in Washington and Benton Counties, Arkansas, between 2015 and 2018. A total of 221 participants were randomly assigned to either a standard or a culturally adapted DSME program. The standard DSME program included 10 h of content delivered across 6 sessions over 6 weeks at a local community health center. In contrast, the adapted DSME program delivered 10 h of content over 8 sessions spanning 8 weeks and was conducted in participants' homes [20].

The standard DSME followed self‐care guidelines established by the American Association of Diabetes Educators and was led by a certified diabetes educator (CDE) with interpreter support. Family members were not required to attend these sessions. The adapted DSME, while informed by the standard curriculum, incorporated culturally relevant modifications to better align with diabetes‐related beliefs and behaviours of Marshallese. This intervention emphasized family involvement, was delivered by a bilingual community health worker (CHW) with a CDE present and included culturally specific components designed to enhance engagement and understanding [20].

To evaluate program effectiveness, clinical measures, including HbA1c, fasting glucose, BMI, fasting lipids, and blood pressure, were collected at baseline, and at 1 week, 6 months, and 12 months post‐intervention. The primary outcome was changes in HbA1c. Additional details on intervention design and study outcomes have been described elsewhere [15, 20]. The DSME trial was approved by the University of Arkansas for Medical Sciences Institutional Review Board, and all participants provided informed consent prior to enrollment.

### 2.3 Model Structure

The Markov model included three primary health states: T2D without complications, T2D‐related complications, and death. The complication states included myocardial infarction (MI), angina, congestive heart failure (CHF), stroke, amputation, impaired vision/blindness (hereafter referred to as blindness), and end‐stage renal disease requiring dialysis (hereafter referred to as dialysis) (Figure S1). These 7 complication states were selected because they represent the principal drivers of long‐term healthcare costs and health outcomes associated with diabetes [21, 22]. By focusing on these clinically significant complications, the model captures the majority of the economic and health burden of T2D while remaining parsimonious and computationally efficient [23].

The simulation began with all individuals in the T2D without complications state, from which they could either remain in that state or transition to a complication state or death over time. Individuals who developed a complication could either remain in that state or transition to death in subsequent cycles. The model assumed each individual could experience only one complication, remaining in that state until the end of a cycle or transitioning to death.

Our simulation population consisted of a hypothetical closed cohort of Marshallese adults with T2D at age 53 years. Sociodemographic and biometric data for people in the initial health state (i.e., T2D without complications) were primarily based on mean baseline values reported in the DSME trial (Table S2) [15, 20] and were used to estimate the risk of diabetes‐related complications. For parameters not available from the trial (e.g., age at T2D diagnosis, smoking status, and serum creatinine level), values were obtained from the published literature [24, 25, 26]. Model assumptions are presented in Table S3. Specifically, we assumed that systolic blood pressure (SBP), BMI, smoking status, and other non‐HbA1c risk factors remained constant throughout the simulation because the DSME trial found no statistically significant changes in BMI, total cholesterol, or high‐density lipoprotein (HDL) cholesterol and did not collect or report data on changes in SBP, smoking status, fasting glucose, low‐density lipoprotein (LDL) cholesterol, or triglycerides [15].

### 2.4 Input Parameters

Table 1 summarizes the parameter estimates, corresponding standard deviation, and assigned probability distributions. When parameter‐specific measures of variability (e.g., standard deviation or 95% confidence interval) were unavailable, upper and lower bounds of uncertainty were specified as ±20% of the mean value [27].

**TABLE 1:** Value, standard deviation, distribution of input parameters.

Parameter	Mean	SD	Distribution	Source
Effect size of the interventions (HbA1c reduction)				
Adapted DSME	−0.87	—	Fixed	15
Standard DSME	−0.08	—	Fixed	15
Transition probabilities^a^				
Myocardial infarction	$1-\exp\left(H\left(t|X_{j}\right)-H\left(t+1|X_{j}\right)\right)$	—	Beta	26
Angina	$1-\exp\left(H\left(t|X_{j}\right)-H\left(t+1|X_{j}\right)\right)$	—	Beta	26
Congestive heart failure	$1-\exp\left(H\left(t|X_{j}\right)-H\left(t+1|X_{j}\right)\right)$	—	Beta	26
Stroke	$1-\exp\left(H\left(t|X_{j}\right)-H\left(t+1|X_{j}\right)\right)$	—	Beta	26
Amputation	$1-\exp\left(H\left(t|X_{j}\right)-H\left(t+1|X_{j}\right)\right)$	—	Beta	26
Blindness	$1-\exp\left(H\left(t|X_{j}\right)-H\left(t+1|X_{j}\right)\right)$	—	Beta	26
End‐stage renal disease with dialysis	$1-\exp\left(H\left(t|X_{j}\right)-H\left(t+1|X_{j}\right)\right)$	—	Beta	26
Costs ($)				
Interventions				
Adapted DSME^b^	1227	125	Gamma	36
Standard DSME^b^	122	12	Gamma	36
Health state				
T2D without complications^b^	4067	415	Gamma	38
Other diseases (first year)				
Myocardial infarction	65215	120694	Gamma	21
Angina	15406	35924	Gamma	21
Congestive heart failure	47026	125200	Gamma	21
Stroke	22060	91433	Gamma	21
Amputation	42731	48415	Gamma	21
Blindness	5251	55893	Gamma	21
End‐stage renal disease with dialysis^b^	110270	11252	Gamma	37
Death (younger than 74)^b^	90050	9189	Gamma	39
Ongoing (subsequent years)				
Myocardial infarction	5939	34199	Gamma	21
Angina	1763	12589	Gamma	21
Congestive heart failure	16895	73436	Gamma	21
Stroke	2248	14213	Gamma	21
Amputation	12164	20441	Gamma	21
Blindness	166	1139	Gamma	21
End‐stage renal disease with dialysis^b^	110270	11252	Gamma	37
Health (dis)utilities^c^				
T2D without complications	0.785	0.053	Beta	22
Myocardial infarction	−0.055	0.006	Beta	22
Angina	−0.09	0.018	Beta	22
Congestive heart failure	−0.108	0.030	Beta	22
Stroke	−0.164	0.029	Beta	22
Amputation	−0.28	0.055	Beta	22
Blindness	−0.074	0.025	Beta	22
End‐stage renal disease with dialysis	−0.175	0.028	Beta	41

Abbreviations: BMI, body mass index; DSME, diabetes self‐management education; HbA1c, haemoglobin A1c; HDL, high‐density lipoprotein; LDL, low‐density lipoprotein; SBP, systolic blood pressure; SD, standard deviation; T2D, type 2 diabetes.

^a^ The corresponding transition probability of T2D‐related complications was calculated as $1-\exp\left(H\left(t|X_{j}\right)-H\left(t+1|X_{j}\right)\right)$, where represents the scale parameter, $X_{j}$ denotes a vector of the risk factors, $\beta_{j}$ represents the associated coefficients, t is the time in the simulation, and ρ is the shape parameter. The risk factors included in the risk estimate of each complication were specified in Table S4.

^b^ The standard deviation was estimated from the mean, with the lower and upper bounds of the 95% confidence interval defined as ±20% of the mean value.

^c^ The standard deviation (σ) was estimated from the 95% confidence interval, assuming a normal distribution, with the lower and upper bounds defined as the mean ±1.96 × σ.

#### 2.4.1 Effect of the DSME Programs

The intervention effect was evaluated based on changes in HbA1c. In the model, a baseline mean HbA1c of 10.46% was applied across all three intervention strategies (adapted DSME, standard DSME, and usual care) [15]. Group‐specific HbA1c values observed at 12‐month post‐intervention (9.59% for adapted DSME; 10.38% for standard DSME) were used to estimate the risk transitioning from the complication‐free T2D state to T2D‐related complication states. No change in risk was assumed for the usual care strategy. Because intervention effects may attenuate over time in the absence of continued intervention, the HbA1c reductions were assumed to persist for 3 years and to return to the baseline level beginning in year 4 of the simulation [28]. This assumption was further examined in the scenario analysis varying the duration of effect (1, 5 and 10 years).

#### 2.4.2 Transition Probabilities

The risk of MI, angina, CHF, stroke, amputation, blindness, and dialysis were estimated using equations developed by Hoerger et al. [26], using pooled longitudinal data on U.S. participants in the Action for Health in Diabetes clinical trial and its follow‐on study as well as the Action to Control Cardiovascular Risk in Diabetes (ACCORD) clinical trial, which continued in the ACCORD Follow‐On Study (ACCORDION). The authors used parametric Weibull hazards models to derive risk equations for each complication, with the cumulative hazard function defined as $H\left(t|X_{j}\right)=\exp\left(\lambda +\beta_{j}X_{j}\right)t^{\rho }$. The corresponding transition probability was calculated as $1-\exp\left(H\left(t|X_{j}\right)-H\left(t+1|X_{j}\right)\right)$, where represents the scale parameter, $X_{j}$ denotes a vector of the risk factors, $\beta_{j}$ represents the associated coefficients, t is the time in the simulation, and ρ is the shape parameter.

These risk equations included several diabetes‐related risk factors, such as HbA1c, SBP, fasting lipid levels, and BMI [26]. Values for these risk factors were obtained directly from the DSME trial whenever available. Values for risk factors not directly available from the DSME trial (e.g., age at diabetes diagnosis, smoking status, serum creatinine levels) were obtained from published sources [24, 25, 26]. Age at diabetes diagnosis was derived from a study by McElfish et al. in which Marshallese individuals reported that the age at which they were informed by the primary care providers that they had diabetes [24]. Smoking prevalence/status was obtained from the World Health Organization estimates of tobacco use among individuals aged 15 years and older in the Marshall Islands [25]. Serum creatinine levels were derived from Hoerger et al. who reported the mean serum creatinine level among U.S. adults with diabetes [26].

Details for the risk factors included in the estimation of each complication are provided in Table S4. Specifically, the risk equation for MI included age at baseline, duration since diabetes diagnosis, sex, education, HbA1c, SBP, LDL cholesterol, HDL cholesterol, triglycerides and smoking status. The angina risk equation included age at baseline, duration since diabetes diagnosis, HbA1c, SBP, LDL cholesterol, HDL cholesterol, triglycerides, BMI, serum creatinine, and ACCORD/ACCORDION participant indicator. The CHF risk equation included age at baseline, duration since diabetes diagnosis, race/ethnicity, education, HbA1c, SBP, HDL cholesterol, BMI, and smoking status. The stroke risk equation included age at baseline, sex, education, HbA1c, SBP, LDL cholesterol, triglycerides, smoking status and ACCORD/ACCORDION participant indicator. The amputation risk equation included age at baseline, duration of diabetes at baseline, sex, race/ethnicity, HbA1c, SBP and smoking status. The blindness risk equation included age at baseline, duration of diabetes at baseline, sex, education, HbA1c, BMI and serum creatinine. Finally, the dialysis risk equation included duration of diabetes, HbA1c, SBP, triglycerides, BMI, and ACCORD/ACCORDION participant indicator.

Mortality varied by health states and was derived from multiple sources. Estimates for end‐stage renal disease requiring dialysis were obtained from a Centers for Disease Control and Prevention report [29], while mortality for MI [30], angina [31], stroke [32] and amputation [33] was based on observational studies. Mortality for CHF was derived from a meta‐analysis [34]. The blindness state was assumed not to confer excess mortality risk, whereas amputation was assumed to increase mortality risk only during the first year of the event, reflecting complications related to the initial surgical procedure [29]. Accordingly, transitions to death for individuals with T2D without complications, those with blindness, and those in the post‐first‐year amputation state were based on the all‐cause mortality among individuals with T2D reported by Gregg et al. [35].

#### 2.4.3 Costs

DSME program costs were obtained from a prior study conducted by our team [36]. In brief, costs per participant were estimated using descriptive analyses that combined total labor costs (e.g., program development, personnel training, session delivery) and non‐labor costs (e.g., printing educational materials) [36].

Costs related to T2D complications were primarily derived from Pelletier et al. [21], where the authors aggregated outpatient, inpatient, and pharmacy claims data. Using International Classification of Diseases codes, the authors estimated complication‐specific costs for two time periods: 1–12 months and 13–24 months following the index date of complication [21]. In our model, costs from the 1–12‐month period were applied as the first‐year (event) costs, while costs from the 13–24‐month period were applied as subsequent‐year (ongoing) costs. Costs for dialysis were derived from a U.S. Renal Data System Annual Data Report [37].

The baseline cost of managing T2D was assumed to remain constant throughout the simulation [38], except upon transition to death. Additionally, we included a one‐time cost of death in the scenario analysis to reflect per capita medical expenditures in the final 12 months of life [39]. All cost estimates were adjusted to 2023 U.S. dollars using the medical care Consumer Price Index (CPI) [40].

#### 2.4.4 Health Utilities

We assigned health‐related quality‐of‐life weight to each health state, using values derived from the EQ‐5D instrument as reported in Clarke et al. [22] and Bagust et al. [41]. The baseline health utility represents the quality of life for individuals with T2D without complications, while transitions to complication states result in utility decrements applied to the baseline value. Once applied, these utility decrements persist for the rest of the simulation unless individuals transition to death.

### 2.5 Data Analysis

#### 2.5.1 Base‐Case Analysis

In the base‐case analysis, we calculated the expected discounted costs and QALYs using the fixed mean value of each input parameter in the simulation. Of note, the resulting aggregate costs and QALYs differed from those generated by the probabilistic sensitivity analysis (PSA), in which input parameter values were randomly sampled from their respective distributions in each iteration. This difference reflects the nonlinear nature of Markov models [42, 43], whereby model outcomes evaluated at the mean parameter values may differ from the mean outcomes obtained by averaging across probabilistic simulations.

Cost‐effectiveness was evaluated using the incremental cost‐effectiveness ratio (ICER) metric and was interpreted as the additional cost required to gain one additional QALY. ICERs were calculated by first ordering the projected QALYs of each strategy in ascending order and then dividing the difference in projected costs by the difference in QALYs between competing strategies. The strategy with an ICER that is below the designated willingness‐to‐pay (WTP) threshold (i.e., $50 000 per QALY) was considered cost‐effective.

#### 2.5.2 Sensitivity Analysis

Deterministic sensitivity analysis (DSA) and PSA were conducted to assess decision uncertainty. The DSA examines the sensitivity of base‐case results to changes in individual input parameters by varying each parameter over a specified and discrete range around its mean value [27]. For costs and utility parameters, 95% confidence intervals were used when available; otherwise, a ±20% range around the mean was assumed. Results of the DSA were summarized using tornado diagrams.

The PSA were conducted to evaluate the cost‐effectiveness of the interventions under joint uncertainty in costs, health utilities, and transition probabilities [27]. In each iteration, parameter values were randomly sampled from their assigned probability distributions. Health utility values and transition probabilities were modelled using beta distributions to reflect their bounded range between 0 and 1, while costs were modelled using gamma distributions to account for right skewness. When measures of variance were not available from the literature, a 20% range around the mean was assumed to approximate the 95% confidence interval [27] and parameterize the corresponding distributions.

PSA results from 1000 iterations were presented using incremental cost‐effectiveness scatterplots to illustrate decision uncertainty at a WTP threshold of $50 000 per QALY gained. This threshold was selected as it has historically served as a benchmark for assessing the value of healthcare interventions in the U.S. [44] Additionally, cost‐effectiveness acceptability curves (CEACs) were generated to depict the probability of each strategy being cost‐effective across a range of WTP thresholds from $0 to $100000 [23].

#### 2.5.3 Scenario Analysis

To examine the robustness of the base‐case analysis results under alternative assumptions, scenario analyses were conducted in which the duration of DSME intervention effects was varied to 1, 5 and 10 years post‐intervention, in addition to the 3‐year duration assumed in the base‐case analysis. In addition, we incorporated a one‐time cost of death for individuals transitioning to the death state.

All analyses were performed using TreeAge Pro Healthcare 2024.

## 3 Results

### 3.1 Base‐Case Analysis

As shown in Table 2, the adapted DSME yielded 0.048 additional QALYs at an incremental cost of $148 compared with standard DSME, resulting in an ICER of $3083/QALY gained. In comparison, standard DSME generated an additional 0.005 QALYs at an incremental cost of $17 relative to usual care, corresponding to an ICER of $3400/QALY gained. Over the 20‐year simulation horizon, the adapted DSME resulted in the highest proportion of individuals remaining in the complication‐free T2D state (20.5%), compared with standard DSME (20.2%) and usual care (20.2%) (Table S5).

**TABLE 2:** Base‐case analysis results.

Strategy	Cost ($)	QALYs	Incremental Cost	Incremental QALYs	ICER ($/QALY)
Adapted DSME	81220	9.272	148	0.048	3083
Standard DSME	81072	9.224	17	0.005	3400
Usual care	81055	9.219	—	—	—

Abbreviations: DSME, diabetes self‐management education; ICER, incremental cost‐effectiveness ratio; QALYs, quality‐adjusted life years.

### 3.2 Sensitivity Analysis

DSA results revealed that base‐case analysis results comparing the adapted DSME with standard DSME were most sensitive to the costs of the adapted DSME program, ongoing dialysis, T2D management, and the standard DSME program (Figure S2).

Figure 1 presents the PSA results from 1000 simulations comparing the adapted DSME program with each alternative strategy at a WTP threshold of $50 000/QALY. At this threshold, the probability that the adapted DSME was cost‐effective relative to standard DSME was 90.2%, and the probability that it was cost‐effective relative to usual care was 93.9%. Figure S3 additionally presents the PSA results on the cost‐effectiveness plane for all three intervention strategies, illustrating the distribution of the simulated cost and effectiveness pairs.

**FIGURE 1:**
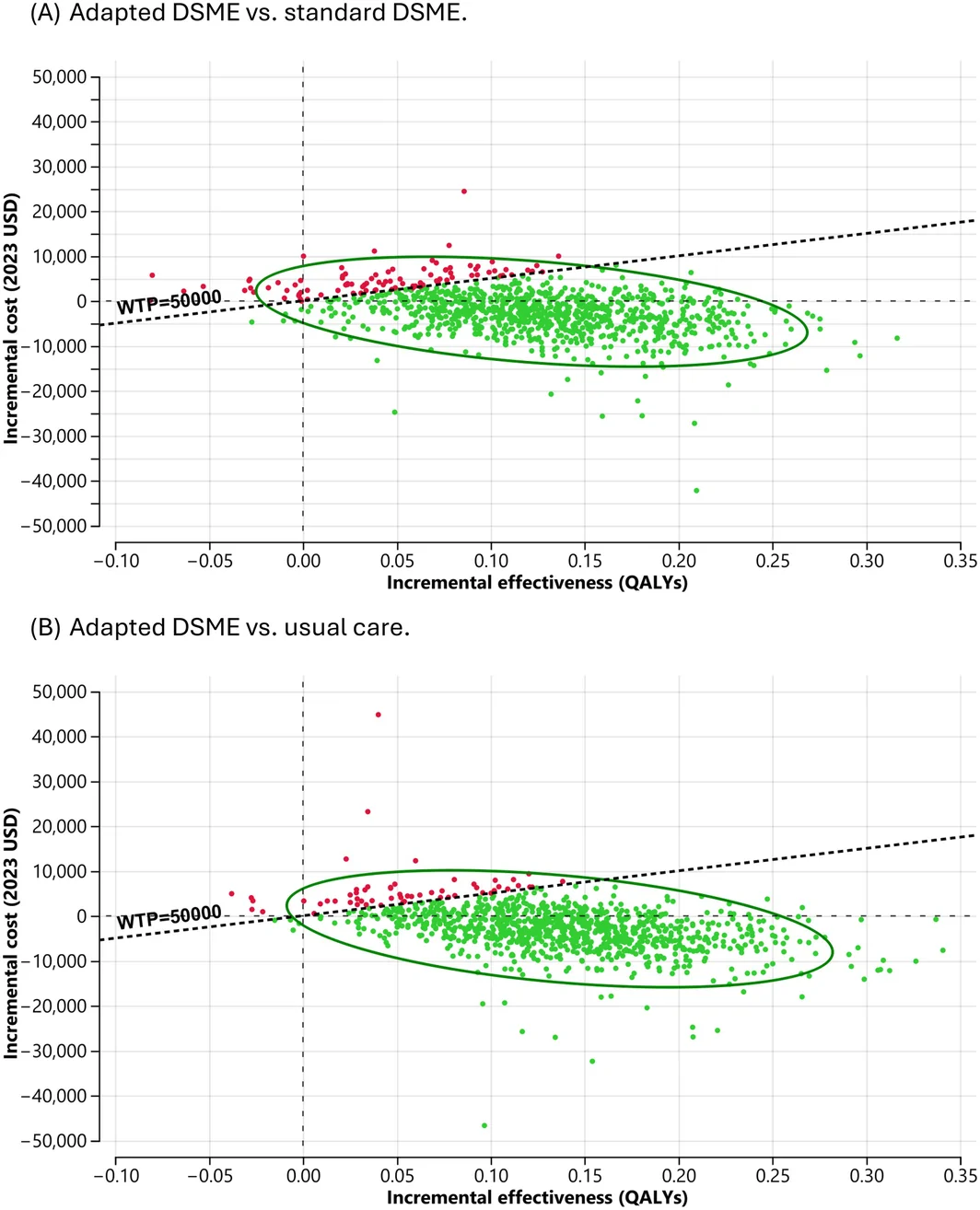
Probabilistic sensitivity analysis results presented as incremental cost‐effectiveness scatterplots. Green dots represent simulation iterations in which the adapted DSME was cost‐effective at a WTP threshold of $50 000 per QALY, whereas red dots indicate iterations exceeding this threshold. DSME, diabetes self‐management education; ICER, incremental cost‐effectiveness ratio; QALY, quality‐adjusted life year; QALYs, quality‐adjusted life years; USD, United States dollar; WTP, willingness‐to‐pay.

CEACs comparing all three strategies showed that the adapted DSME program was the preferred strategy in 87.1% and 93.7% of simulations at WTP thresholds of $50 000 and $100000 per QALY, respectively. In comparison, standard DSME was favoured in 8.4% and 4.1% of simulations, and usual care in 4.5% and 2.2% of simulations at the same thresholds (Figure 2).

**FIGURE 2:**
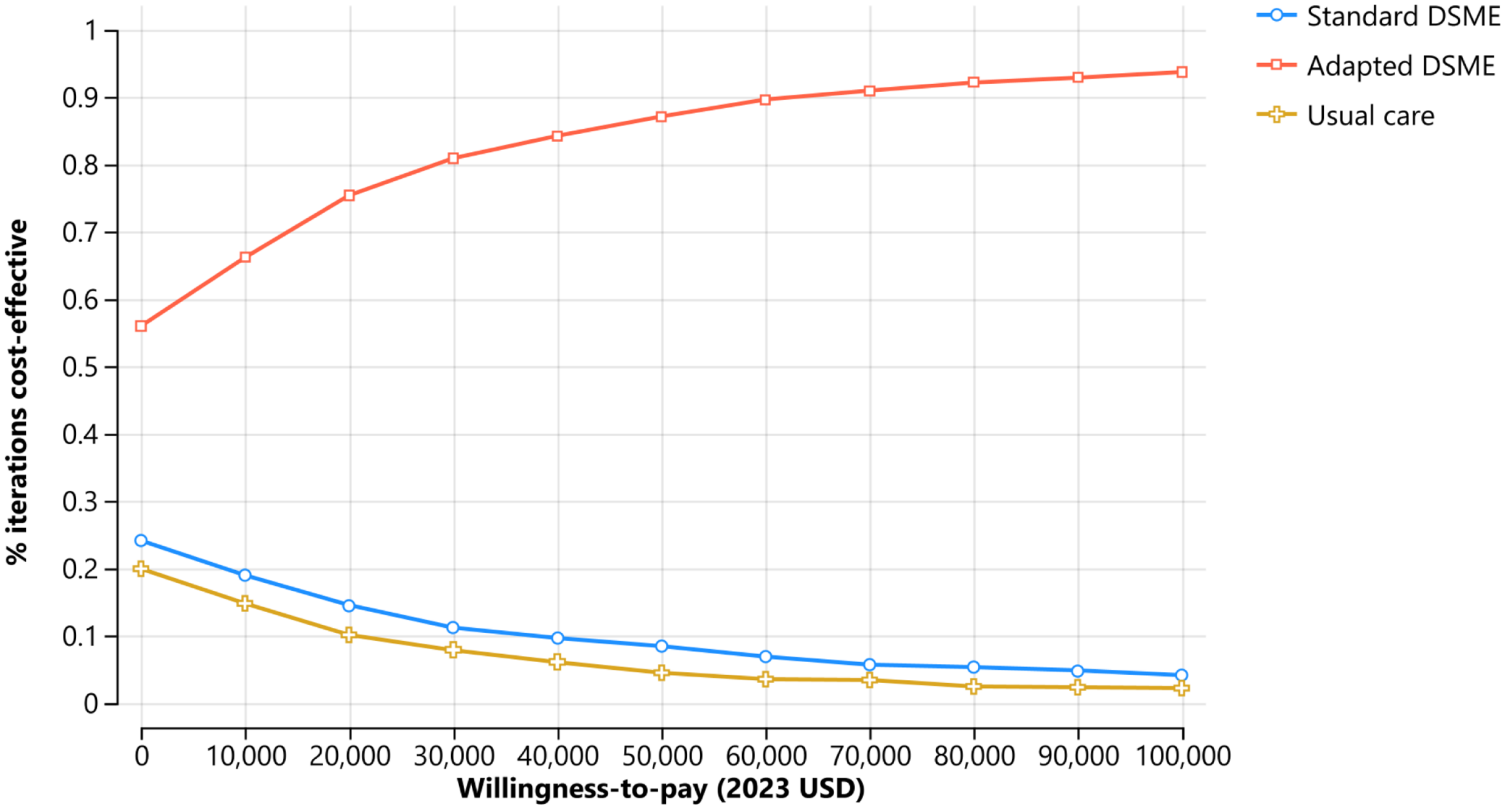
Cost‐effectiveness acceptability curve. DSME, diabetes self‐management education; USD, United States dollar.

### 3.3 Scenario Analysis

Both DSME strategies demonstrated greater effectiveness and lower costs as the assumed duration of the intervention effect increased. Under the 5‐ and 10‐year effect duration scenarios, the adapted DSME dominated both the standard DSME and usual care (Table 3).

**TABLE 3:** Scenario analysis results on the assumption of intervention effect (changes in HbA1c levels) duration.

Assumption/strategy	Cost ($)	QALYs	Incremental cost	Incremental QALYs	ICER ($/QALY)
Diminished intervention effects after 1 year					
Adapted DSME	81874	9.238	737	0.017	43353
Standard DSME	81137	9.221	82	0.002	41000
Usual care	81055	9.219	—	—	—
Diminished intervention effects after 5 years					
Adapted DSME	80702	9.300	−320	0.073	Cost‐saving
Standard DSME	81022	9.227	−33	0.008	Cost‐saving
Usual care	81055	9.219	—	—	—
Diminished intervention effects after 10 years					
Adapted DSME	79717	9.346	−1216	0.114	Cost‐saving
Standard DSME	80933	9.232	−122	0.013	Cost‐saving
Usual care	81055	9.219	—	—	—
Base model using cost of death					
Adapted DSME	117235	9.272	−197	0.048	Cost‐saving
Standard DSME	117432	9.224	−21	0.005	Cost‐saving
Usual care	117453	9.219	—	—	—

Abbreviations: DSME, diabetes self‐management education; ICER, incremental cost‐effectiveness ratio; QALYs, quality‐adjusted life years.

In the 1‐year effect duration scenario, the adapted DSME generated an additional 0.017 QALYs at an incremental cost of $737 relative to standard DSME, yielding an ICER of $43353 per QALY. The standard DSME produced an additional 0.002 QALYs at an incremental cost of $82 compared with usual care, resulting in an ICER of $41000 per QALY. When a one‐time cost of death was incorporated, the adapted DSME became cost‐saving compared with the standard DSME, while the standard DSME became cost‐saving when compared with usual care (Table 3).

## 4 Discussion

We assessed the health and economic consequences of a culturally adapted, family DSME program for Marshallese adults with T2D. Simulation results demonstrated that a culturally adapted DSME program was the preferred strategy, yielding either cost‐savings (i.e., greater QALYs at lower costs) or being cost‐effective compared with both standard DSME and usual care. The long‐term incremental health benefits and cost savings were relatively modest, reflecting the small effect size of the interventions. Accordingly, these findings should be interpreted with caution. Nevertheless, sensitivity and scenario analyses demonstrated that the conclusions were robust across a range of plausible assumptions. These findings suggest that culturally tailored diabetes education, aligned with the social and cultural context of target audiences, may enhance engagement and improve health outcomes [15, 45, 46].

To the best of our knowledge, few CEAs of culturally adapted DSME programs have been conducted. One prior study used RCT data among uninsured Mexican Americans and reported an ICER of $355 per QALY gained over a 20‐year horizon for a culturally tailored intervention involving CHW support compared to usual care [17]. Of note, the intervention was cost‐saving among participants aged 55–75 years [17]. These findings are consistent with our results, in which the adapted DSME was the preferred strategy over a 20‐year horizon in a simulated population with a mean baseline age of 53 years.

Our findings have important implications for both the Marshallese and the broader health care system. Although data on diabetes‐related health care utilization and expenditures among Marshallese populations are limited, the average annual medical cost for individuals with diabetes in the U.S. is approximately $20000, contributing to total national direct costs exceeding $300 billion [3]. Given the observed improvements in HbA1c associated with the adapted DSME, such programs may reduce the incidence of diabetes‐related complications and, in turn, alleviate the financial burden on both patients and the healthcare system.

These results also support expanding reimbursements for culturally adapted DSME programs among both public and private payers. A 2015 joint statement by the American Diabetes Association (ADA), the Association of Diabetes Care & Education Specialists (ADCES), and the Academy of Nutrition and Dietetics highlighted the importance of initiating DSME services at the time of diagnosis [47]. However, the long‐term sustainability of these programs is closely tied to consistent and adequate insurance coverage. Although Medicare reimburses DSME services under Medicare Part B when the services are ADA‐recognized or ADCES‐accredited [48], coverage across private insurers and Medicaid programs remains inconsistent. As of recent reports, 17 states did not require DSME coverage, only 15 mandate Medicaid coverage, and 18 provide partial or conditional coverage through sub‐regulatory Medicaid guidance [49], including Medicaid expansions in several states which represent meaningful progress toward improving access to DSME services. For example, states such as Colorado and Mississippi have expanded DSME coverage within their Medicaid programs [50]. Nonetheless, persistent gaps in coverage highlight the need for more uniform reimbursement policies to ensure equitable access to culturally tailored DSME interventions.

The progress in reimbursement policy for DSME may be particularly impactful for the Marshallese population. As migrants under the Compact of Free Association (COFA), Marshallese individuals were historically excluded from Medicaid and Medicaid expansion benefits following the enactment of the Personal Responsibility and Work Opportunity Reconciliation Act of 1996 [51, 52]. Although they remained eligible to purchase private insurance [52], prior estimates indicated that approximately half of Marshallese living in the U.S. were uninsured [4, 6]. This lack of coverage has posed a substantial barrier to accessing essential diabetes care services, including DSME. The passage of the Consolidated Appropriations Act of 2021 marked a critical policy shift by restoring Medicaid eligibility for COFA migrants [53]. This change has important implications for access to DSME, as insurance coverage is a well‐documented facilitator of DSME participation [54]. Consequently, expanded Medicaid eligibility may increase DSME uptake among Marshallese with T2D.

This study contributes to the limited body of literature on economic evaluations of culturally adapted DSME programs and, to our knowledge, represents the first CEA of DSME interventions among Marshallese adults with T2D. The disproportionate burden of diabetes and suboptimal self‐management behaviours observed in this population highlight the importance of targeted interventions aimed at improving health outcomes while reducing long‐term healthcare costs. In addition, the risk equations used to project T2D‐related complications were derived from recent U.S.‐based clinical trials and real‐world settings, offering contemporary and contextually relevant evidence on the association between glycemic control and T2D‐related outcomes.

### 4.1 Limitations

Several limitations should be noted. First, because the Marshallese population is relatively understudied in the U.S., some model parameters may not fully reflect the characteristics of the study population. For example, validated risk equations for predicting diabetes‐related complications in Marshallese adults are unavailable. Although many diabetes simulation models rely on equations developed from the United Kingdom Prospective Diabetes Study [55], those equations were derived from individuals with newly diagnosed T2D in the United Kingdom beginning in the late 1970s. Instead, we applied risk equations developed from more recent large U.S. clinical trials, which may better represent Marshallese adults residing in the U.S. Similarly, cost estimates for diabetes‐related complications specific to Marshallese adults are unavailable; therefore, we applied estimates derived from U.S. data to maximize the relevance of the analysis. Nevertheless, future research is needed to develop or calibrate risk equations for diabetes‐related complications using data from Marshallese adults or other similar Pacific Islander populations, as population‐specific risks may differ from those observed in the general U.S. population.

In addition, we assumed that effects for both DSME programs persisted for 3 years in the base‐case analysis. The DSME trial where our data were derived followed participants for only 12 months after the intervention, and no studies have evaluated the long‐term persistence of intervention effects among Marshallese adults with T2D. Consequently, we adopted a 3‐year duration based on a previous diabetes simulation study [28]. To address the uncertainty associated with transferring these parameters and assumptions, we conducted extensive sensitivity and scenario analyses using wide parameter ranges and multiple plausible assumptions.

Second, risk factors for diabetes‐related complications were assumed to remain constant over the simulation horizon, which may not accurately reflect real‐world disease progression or changes in individual health status over time. Third, individuals were restricted to transitioning to only one complication state during the simulation. Although this approach balanced model realism with computational feasibility, it may not fully capture the cumulative disease burden and interactions of multiple diabetes‐related complications experienced over time. Fourth, the risk equations used to predict T2D‐related complications were derived from a study with a maximum follow‐up of 13 years [26]. Although this limitation is common in simulation modelling, the underlying cohorts included individuals aged 45–75 years who had often lived with T2D for at least 10 years, making them broadly comparable to the population modelled in this study [26].

Fifth, we did not evaluate the assumption that SBP, BMI, smoking status, and other non‐HbA1c risk factors remained constant throughout the simulation in the sensitivity analyses because the DSME trial did not provide evidence of intervention‐related changes in these risk factors. Allowing these parameters to vary without empirical support would have required unsupported assumptions regarding the magnitude and direction of change. Consequently, our findings reflect the estimated long‐term impact attributable to changes in HbA1c alone and may therefore underestimate the intervention's overall benefits if future evidence demonstrates meaningful improvements in other cardiovascular risk factors.

Finally, several variables required for the risk equations (e.g., age at diabetes diagnosis, smoking status, and serum creatinine levels) were not collected in the DSME trial and were therefore obtained from published literature [24, 25, 26]. The use of externally sourced inputs may have introduced additional uncertainty and influenced the simulation results.

## 5 Conclusion

The culturally adapted DSME program for Marshallese adults with T2D is a cost‐effective intervention over a 20‐year time horizon, although the results should be interpreted with caution because the differences in costs and health outcomes between the intervention strategies were modest. Nevertheless, these findings support the potential value of culturally tailored diabetes self‐management education programs and provide evidence to inform reimbursement decisions by public and private health insurers.

## Author Contributions

Conceptualization: D.L.‐C., H.W., S.‐H.C., P.A.M., T.L.M. Formal analysis: D.L.‐C. Visualization: D.L.‐C. Methodology: D.L.‐C., H.W., T.L.M. Writing‐original draft: D.L.‐C., T.L.M. Writing‐review and editing: D.L.‐C., H.W., S.‐H.C., P.A.M., J.A.A., T.L.M. Supervision: T.L.M.

## Funding

The authors have nothing to report.

## Conflicts of Interest

The authors declare no conflicts of interest.

## Supporting information


**Table S1:** CHEERS 2022 Checklist.
**Table S2:** Simulation population baseline characteristics.
**Table S3:** Simulation model assumptions.
**Table S4:** Risk factors included in the transition probability estimation of T2D‐related complications.
**Table S5:** Markov trace results of the base‐care analysis.
**Figure S1:** Schematic overview of disease progression depicting health states.
**Figure S2:** Tornado diagram depicting one‐way discrete sensitivity analysis results comparing adapted DSME versus standard DSME. DSME, diabetes self‐management education; QALY, quality‐adjusted life year; T2D, type 2 diabetes; USD, United States dollar.
**Figure S3:** Cost‐effectiveness plane depicting probabilistic sensitivity analysis results for each strategy. Each point represents one simulation iteration's costs and effectiveness. DSME, diabetes self‐management education; QALY, quality‐adjusted life year; USD, United States dollar.

## Data Availability

The data that support the findings of this study are available from the corresponding author upon request.
