# Supplementary material for: Cost‐Effectiveness of a Culturally Adapted Diabetes Self‐Management Education Program for Marshallese Adults With Type 2 Diabetes‐a State‐Transition Markov Analysis
Source: Diabetes Obes Metab. 2026 Sep 17;28(12):11556–67. doi: 10.1111/dom.71351 (PMC13730993; doi:10.1111/dom.71351)
Supplement: Supplementary file 1 [file DOM-28-11556-s001.docx]

**Appendix Table S1. CHEERS 2022 Checklist.**

|  | **Item** | **Guidance for Reporting** | **Reported in Section** |
| --- | --- | --- | --- |
| **TITLE** | | |  |
| Title | 1 | Identify the study as an economic evaluation and specify the Interventions being compared. | Title/page 1 |
| **ABSTRACT** | | |  |
| Abstract | 2 | Provide a structured summary that highlights context, key Methods, results and alternative analyses. | Abstract/pages 2-3 |
| **INTRODUCTION** | | |  |
| Background and objectives | 3 | Give the context for the study, the study question and its practical relevance for decision making in policy or practice. | Introduction/ pages 4-5 |
| **METHODS** | | |  |
| Health economic analysis plan | 4 | Indicate whether a health economic analysis plan was developed and where available. | N/A |
| Study population | 5 | Describe characteristics of the study population (such as age range,  demographics, socioeconomic, or clinical characteristics). | Methods/pages 5-8 |
| Setting and location | 6 | Provide relevant contextual information that may influence findings. | Methods/pages 5-8 |
| Comparators | 7 | Describe the interventions or strategies being compared and why chosen. | Methods/pages 5-8 |
| Perspective | 8 | State the perspective(s) adopted by the study and why chosen. | Methods/page 6 |
| Time horizon | 9 | State the time horizon for the study and why appropriate. | Methods/page 6 |
| Discount rate | 10 | Report the discount rate(s) and reason chosen. | Methods/page 6 |
| Selection of outcomes | 11 | Describe what outcomes were used as the measure(s) of benefit(s) and harm(s). | Methods/pages 9-13 |
| Measurement of  outcomes | 12 | Describe how outcomes used to capture benefit(s) and harm(s) were measured. | Methods/pages 9-13 |
| Valuation of outcomes | 13 | Describe the population and methods used to measure and value outcomes. | Methods/pages 8-13 |
| Measurement and  valuation of resources and costs | 14 | Describe how costs were valued. | Methods/pages 11-12 |
| Currency, price date, and conversion | 15 | Report the dates of the estimated resource quantities and unit costs, plus the currency and year of conversion. | Methods/page 12 |
| Rationale and description of model | 16 | If modelling is used, describe in detail and why used. Report if the model is publicly available and where it can be accessed. | Methods/pages 7-8  and Appendix |
| Analytics and assumptions | 17 | Describe any methods for analyzing or statistically transforming data, any extrapolation methods, and approaches for validating any model used. | N/A |
| Characterizing heterogeneity | 18 | Describe any methods used for estimating how the results of the study vary for sub-groups. | N/A |
| Characterizing  distributional effects | 19 | Describe how impacts are distributed across different individuals or adjustments made to reflect priority populations. | N/A |
| Characterizing  uncertainty | 20 | Describe methods to characterize any sources of uncertainty in the analysis. | Methods/pages  13-15 |
| Approach to engagement with patients and others affected by the study | 21 | Describe any approaches to engage patients or service recipients, the general public, communities, or stakeholders (e.g., clinicians or payers) in the design of the study. | N/A |
| **RESULTS** | | |  |
| Study parameters | 22 | Report all analytic inputs (e.g., values, ranges, references) including  uncertainty or distributional assumptions. | Methods/pages 8-15 and Table 1 |
| Summary of main results | 23 | Report the mean values for the main categories of costs and outcomes of interest and summarize them in the most appropriate overall measure. | Results/page 15 |
| Effect of uncertainty | 24 | Describe how uncertainty about analytic judgments, inputs, or projections affect findings. Report the effect of choice of discount rate and time horizon, if applicable. | Results/pages 15-17 |
| Effect of engagement  with patients and others affected by the study | 25 | Report on any difference patient/service recipient, general public,  community, or stakeholder involvement made to the approach or findings of the study. | N/A |
| **DISCUSSION** | | |  |
| Study findings, limitations, generalizability, and current knowledge | 26 | Report key findings, limitations, ethical or equity considerations not captured, and how these could impact patients, policy, or practice. | Discussion/pages 17-22 |
| **OTHER RELEVANT INFORMATION** | | |  |
| Source of funding | 27 | Describe how the study was funded and any role of the funder in the identification, design, conduct, and reporting of the analysis. | Funding information  statement/page 23 |
| Conflicts of interest | 28 | Report authors conflicts of interest according to journal or International Committee of Medical Journal Editors requirements. | Conflict of interest statement/page 23 |

**Appendix Table S2**. Simulation population baseline characteristics.

| Characteristics | Value | Source |
| --- | --- | --- |
| Hemoglobin A1c (%)^†^ | 10.46 | 15 |
| Age of diabetes diagnosis (years)^‡^ | 43 | 24 |
| Age at beginning of simulation (years) | 53 | 15 |
| Female (%) | 58.82 | 15 |
| Education: more than high school (%) | 9.95 | 15 |
| Body mass index (kg/m^2^) | 30.99 | 15 |
| Systolic blood pressure (mm Hg) | 134.23 | 15 |
| Low-density lipoprotein cholesterol (mg/dL) | 108.75 | 15 |
| High-density lipoprotein cholesterol (mg/dL) | 36.91 | 15 |
| Triglycerides (mg/dL) | 233.63 | 15 |
| Smoking (%)^§^ | 29.10 | 25 |
| Serum creatinine (mg/dL)^¶^ | 0.94 | 26 |

† Applied across 3 strategies in the simulation model.

‡ Age of diabetes diagnosis was estimated from Marshallese individuals’ reported age they were told they had diabetes.

§ Smoking was estimated using prevalence of tobacco use in the Marshall Islands.

¶ Serum creatinine was estimated from an average value of adults with T2D from the National Health and Nutrition Examination Survey (NHANES).

**Appendix Table S3**. Simulation model assumptions.

| **Assumptions** | **Source** |
| --- | --- |
| 1. Individuals have T2D without complications at the beginning of the simulation | 29,39 |
| 2. Sociodemographic and biometric data unavailable from the DSME randomized controlled trial were obtained from the literature and assumed to be applicable to our simulation population | 24-26 |
| 3. The intervention effects of the DSME programs persisted for 3 years | 28 |
| 4. Individual risk factors (e.g. smoking prevalence, body mass index, systolic blood pressure) were held constant in simulation | Authors |
| 5. Patients in the myocardial infarction, angina, congestive heart failure, stroke, dialysis, and first-year amputation health states have excess mortality risk compared with those without complications | 29,39 |
| 6. A baseline cost of managing T2D persisted for individuals who did not transition to the death state | 39 |
| 7. Complication costs consisted of an initial cost in the first year of onset and subsequent annual costs associated with ongoing management of complications | 29,39 |
| 8. The utility decrement persisted after entry into a complication state unless a transition to death occurred | 29,39 |
| 9. Standard deviations for costs and utilities were estimated from reported mean values and confidence intervals when not obtainable in the literature | 27 |

DSME, diabetes self-management education; HbA1c, hemoglobin A1c; T2D, type 2 diabetes.

**Appendix Table S4**. Risk factors included in the transition probability estimation of T2D-related complications.

| Complication^†,§^ | Risk factors ($\boldsymbol{X}_{\boldsymbol{j}}$)^26‡^ |
| --- | --- |
| Myocardial infarction | Age at baseline, duration since diabetes diagnosis, sex, education, HbA1c, SBP, LDL cholesterol, HDL cholesterol, ln(triglycerides), smoking status |
| Angina | Age at baseline, duration since diabetes diagnosis, HbA1c, SBP, LDL cholesterol, HDL cholesterol, ln(triglycerides), BMI, serum creatinine, ACCORD/ACCORDION participant |
| Congestive heart failure | Age at baseline, duration since diabetes diagnosis, race/ethnicity, education, HbA1c, SBP, HDL cholesterol, BMI, smoking status |
| Stroke | Age at baseline, sex, education, HbA1c, SBP, LDL cholesterol, ln(triglycerides), smoking status, ACCORD/ACCORDION participant |
| Amputation | Age at baseline, duration of diabetes at baseline, sex, race/ethnicity, HbA1c, SBP, smoking status |
| Blindness | Age at baseline, duration of diabetes at baseline, sex, education, HbA1c, BMI, serum creatinine |
| Dialysis | Duration of diabetes at baseline, HbA1c, SBP, ln(triglycerides), BMI, ACCORD/ACCORDION participant |

T2D, type 2 diabetes; ACCORD/ACCORDION, Action to Control Cardiovascular Risk in Diabetes; BMI, body mass index; HbA1c, hemoglobin A1c; HDL, high-density lipoprotein; LDL, low-density lipoprotein; SBP, systolic blood pressure.

† The duration since diabetes diagnosis was modeled as a time-varying variable, measured continuously from the date of diabetes diagnosis; whereas the duration of diabetes at baseline was a fixed variable representing the number of years between an individual's diabetes diagnosis and the start of the simulation.

‡ The cumulative hazard function defined as $H(t{|X}_{j})=exp(\lambda+\beta_{j}X_{j})t^{\rho}$. The corresponding transition probability for transition probability was calculated as $1-exp(H\left( t | X_{j} \right)-H\left( t+1 | X_{j} \right))$, where $\lambda$ represents the scale parameter,$X_{j}$denotes a vector of the risk factors, $\beta_{j}$represents the associated coefficients, $t$ is the time in the simulation, and $\rho$ is the shape parameter.

§ We included an ACCORD/ACCORDION participant indicator (=1) in the angina, stroke, and dialysis risk equations, as ACCORD/ACCORDION comprises individuals with established type 2 diabetes who are at elevated risk for cardiovascular disease, making this cohort more representative of the Marshallese patients included in the simulation. Additionally, this approach was more conservative, as it accounts for the possibility that the simulated population may be at higher risk for certain diabetes-related complications.

**Appendix Table S5**. Markov trace results of the base-care analysis.

(A) Adapted DSME.

| Cycle | T2D without complications | Blindness | Dialysis | Amputation | MI | Stroke | CHF | Angina | Death |
| --- | --- | --- | --- | --- | --- | --- | --- | --- | --- |
| 0 | 100.0% | 0.0% | 0.0% | 0.0% | 0.0% | 0.0% | 0.0% | 0.0% | 0.0% |
| 1 | 95.0% | 0.8% | 0.5% | 0.0% | 1.1% | 0.5% | 0.1% | 0.6% | 1.4% |
| 2 | 90.0% | 1.6% | 0.9% | 0.1% | 2.0% | 0.9% | 0.2% | 1.2% | 3.2% |
| 3 | 85.1% | 2.4% | 1.2% | 0.1% | 2.9% | 1.2% | 0.3% | 1.6% | 5.1% |
| 4 | 79.8% | 3.2% | 1.5% | 0.1% | 3.8% | 1.7% | 0.4% | 2.1% | 7.3% |
| 5 | 74.7% | 4.1% | 1.7% | 0.2% | 4.6% | 2.0% | 0.5% | 2.5% | 9.8% |
| 6 | 69.7% | 4.9% | 1.8% | 0.2% | 5.2% | 2.3% | 0.7% | 2.9% | 12.4% |
| 7 | 64.9% | 5.6% | 1.8% | 0.3% | 5.8% | 2.5% | 0.8% | 3.2% | 15.1% |
| 8 | 60.3% | 6.3% | 1.8% | 0.3% | 6.3% | 2.6% | 0.9% | 3.4% | 18.0% |
| 9 | 55.9% | 7.0% | 1.8% | 0.3% | 6.6% | 2.7% | 1.0% | 3.6% | 21.0% |
| 10 | 51.7% | 7.5% | 1.7% | 0.3% | 7.0% | 2.8% | 1.1% | 3.8% | 24.0% |
| 11 | 47.7% | 8.1% | 1.7% | 0.3% | 7.2% | 2.8% | 1.2% | 4.0% | 27.1% |
| 12 | 43.9% | 8.5% | 1.6% | 0.4% | 7.4% | 2.8% | 1.3% | 4.1% | 30.2% |
| 13 | 40.3% | 9.0% | 1.5% | 0.4% | 7.5% | 2.7% | 1.3% | 4.2% | 33.2% |
| 14 | 36.9% | 9.3% | 1.3% | 0.4% | 7.5% | 2.6% | 1.4% | 4.3% | 36.3% |
| 15 | 33.7% | 9.6% | 1.2% | 0.4% | 7.6% | 2.6% | 1.4% | 4.3% | 39.3% |
| 16 | 30.7% | 9.8% | 1.1% | 0.4% | 7.5% | 2.5% | 1.4% | 4.3% | 42.3% |
| 17 | 27.9% | 10.0% | 1.0% | 0.4% | 7.5% | 2.4% | 1.4% | 4.4% | 45.1% |
| 18 | 25.2% | 10.1% | 0.9% | 0.3% | 7.4% | 2.3% | 1.4% | 4.3% | 48.0% |
| 19 | 22.8% | 10.2% | 0.8% | 0.3% | 7.3% | 2.1% | 1.4% | 4.3% | 50.7% |
| 20 | 20.5% | 10.3% | 0.7% | 0.3% | 7.1% | 2.0% | 1.4% | 4.3% | 53.4% |

DSME, diabetes self-management education; T2D, type 2 diabetes; CHF, congestive heart failure; dialysis, end-stage renal disease with dialysis; MI, myocardial infarction.

(B) Standard DSME.

| Cycle | T2D without complications | Blindness | Dialysis | Amputation | MI | Stroke | CHF | Angina | Death |
| --- | --- | --- | --- | --- | --- | --- | --- | --- | --- |
| 0 | 100.0% | 0.0% | 0.0% | 0.0% | 0.0% | 0.0% | 0.0% | 0.0% | 0.0% |
| 1 | 94.5% | 0.8% | 0.6% | 0.0% | 1.2% | 0.6% | 0.1% | 0.7% | 1.4% |
| 2 | 89.1% | 1.7% | 1.1% | 0.1% | 2.3% | 1.1% | 0.2% | 1.3% | 3.2% |
| 3 | 83.8% | 2.5% | 1.4% | 0.1% | 3.3% | 1.5% | 0.3% | 1.8% | 5.3% |
| 4 | 78.6% | 3.4% | 1.6% | 0.2% | 4.1% | 1.9% | 0.4% | 2.3% | 7.6% |
| 5 | 73.5% | 4.2% | 1.8% | 0.2% | 4.8% | 2.2% | 0.6% | 2.7% | 10.1% |
| 6 | 68.6% | 5.0% | 1.9% | 0.3% | 5.5% | 2.4% | 0.7% | 3.0% | 12.8% |
| 7 | 63.9% | 5.7% | 1.9% | 0.3% | 6.0% | 2.6% | 0.8% | 3.3% | 15.6% |
| 8 | 59.3% | 6.4% | 1.9% | 0.3% | 6.4% | 2.7% | 0.9% | 3.5% | 18.5% |
| 9 | 55.0% | 7.0% | 1.9% | 0.3% | 6.8% | 2.8% | 1.0% | 3.8% | 21.5% |
| 10 | 50.9% | 7.6% | 1.8% | 0.4% | 7.1% | 2.8% | 1.1% | 3.9% | 24.5% |
| 11 | 46.9% | 8.1% | 1.7% | 0.4% | 7.3% | 2.8% | 1.2% | 4.1% | 27.6% |
| 12 | 43.2% | 8.6% | 1.6% | 0.4% | 7.4% | 2.8% | 1.3% | 4.2% | 30.7% |
| 13 | 39.6% | 9.0% | 1.5% | 0.4% | 7.5% | 2.7% | 1.3% | 4.3% | 33.8% |
| 14 | 36.3% | 9.3% | 1.3% | 0.4% | 7.6% | 2.6% | 1.4% | 4.3% | 36.8% |
| 15 | 33.1% | 9.6% | 1.2% | 0.4% | 7.6% | 2.6% | 1.4% | 4.4% | 39.8% |
| 16 | 30.2% | 9.8% | 1.1% | 0.4% | 7.6% | 2.5% | 1.4% | 4.4% | 42.7% |
| 17 | 27.4% | 10.0% | 1.0% | 0.4% | 7.5% | 2.4% | 1.4% | 4.4% | 45.6% |
| 18 | 24.8% | 10.1% | 0.9% | 0.4% | 7.4% | 2.2% | 1.4% | 4.4% | 48.4% |
| 19 | 22.4% | 10.2% | 0.8% | 0.3% | 7.3% | 2.1% | 1.4% | 4.4% | 51.1% |
| 20 | 20.2% | 10.2% | 0.7% | 0.3% | 7.1% | 2.0% | 1.4% | 4.3% | 53.8% |

DSME, diabetes self-management education; T2D, type 2 diabetes; CHF, congestive heart failure; dialysis, end-stage renal disease with dialysis; MI, myocardial infarction.

(C) Usual Care.

| Cycle | T2D without complications | Blindness | Dialysis | Amputation | MI | Stroke | CHF | Angina | Death |  |
| --- | --- | --- | --- | --- | --- | --- | --- | --- | --- | --- |
| 0 | 100.0% | 0.0% | 0.0% | 0.0% | 0.0% | 0.0% | 0.0% | 0.0% | 0.0% | |
| 1 | 94.5% | 0.8% | 0.6% | 0.0% | 1.3% | 0.6% | 0.1% | 0.7% | 1.4% | |
| 2 | 89.0% | 1.7% | 1.1% | 0.1% | 2.4% | 1.1% | 0.2% | 1.3% | 3.2% | |
| 3 | 83.6% | 2.5% | 1.4% | 0.1% | 3.3% | 1.5% | 0.3% | 1.9% | 5.3% | |
| 4 | 78.4% | 3.4% | 1.7% | 0.2% | 4.2% | 1.9% | 0.4% | 2.3% | 7.6% | |
| 5 | 73.4% | 4.2% | 1.8% | 0.2% | 4.9% | 2.2% | 0.6% | 2.7% | 10.1% | |
| 6 | 68.5% | 5.0% | 1.9% | 0.3% | 5.5% | 2.4% | 0.7% | 3.0% | 12.8% | |
| 7 | 63.8% | 5.7% | 1.9% | 0.3% | 6.0% | 2.6% | 0.8% | 3.3% | 15.6% | |
| 8 | 59.2% | 6.4% | 1.9% | 0.3% | 6.4% | 2.7% | 0.9% | 3.6% | 18.5% | |
| 9 | 54.9% | 7.0% | 1.9% | 0.3% | 6.8% | 2.8% | 1.0% | 3.8% | 21.5% | |
| 10 | 50.8% | 7.6% | 1.8% | 0.4% | 7.1% | 2.8% | 1.1% | 3.9% | 24.6% | |
| 11 | 46.8% | 8.1% | 1.7% | 0.4% | 7.3% | 2.8% | 1.2% | 4.1% | 27.7% | |
| 12 | 43.1% | 8.6% | 1.6% | 0.4% | 7.5% | 2.8% | 1.3% | 4.2% | 30.7% | |
| 13 | 39.6% | 9.0% | 1.5% | 0.4% | 7.5% | 2.7% | 1.3% | 4.3% | 33.8% | |
| 14 | 36.2% | 9.3% | 1.3% | 0.4% | 7.6% | 2.6% | 1.4% | 4.3% | 36.8% | |
| 15 | 33.1% | 9.6% | 1.2% | 0.4% | 7.6% | 2.6% | 1.4% | 4.4% | 39.8% | |
| 16 | 30.1% | 9.8% | 1.1% | 0.4% | 7.6% | 2.5% | 1.4% | 4.4% | 42.8% | |
| 17 | 27.4% | 10.0% | 1.0% | 0.4% | 7.5% | 2.3% | 1.4% | 4.4% | 45.7% | |
| 18 | 24.8% | 10.1% | 0.9% | 0.4% | 7.4% | 2.2% | 1.4% | 4.4% | 48.5% | |
| 19 | 22.4% | 10.2% | 0.8% | 0.3% | 7.3% | 2.1% | 1.4% | 4.4% | 51.2% | |
| 20 | 20.2% | 10.2% | 0.7% | 0.3% | 7.1% | 2.0% | 1.4% | 4.3% | 53.8% | |

DSME, diabetes self-management education; T2D, type 2 diabetes; CHF, congestive heart failure; dialysis, end-stage renal disease with dialysis; MI, myocardial infarction.

**Appendix Figure S1**. Schematic overview of disease progression depicting health states.

T2D, type 2 diabetes; CHF, congestive heart failure; MI, myocardial infarction.

**Appendix Figure S2**. Tornado diagram depicting one-way discrete sensitivity analysis results comparing adapted DSME vs. standard DSME. DSME, diabetes self-management education; QALY, quality-adjusted life year; T2D, type 2 diabetes; USD, United States dollar.

**Appendix Figure S3**. Cost-effectiveness plane depicting probabilistic sensitivity analysis results for each strategy. Each point represents one simulation iteration’s costs and effectiveness. DSME, diabetes self-management education; QALY, quality-adjusted life year; USD, United States dollar.
